# Supplementary material for: Exploration on Optimized Control Way of D-Amino Acid for Efficiently Mitigating Membrane Biofouling of Membrane Bioreactor
Source: Membranes (Basel). 2021 Aug 11;11(8):612. doi: 10.3390/membranes11080612 (PMC8401574; doi:10.3390/membranes11080612)
Supplement: Supplementary file 1 [file membranes-11-00612-s001.zip › membranes-1311780-supplementary.pdf]

Copyright

Supporting Information

Exploration on optimized control way of D-amino acid for efficiently mitigating membrane biofouling of membrane bioreactor

*Zhan Gao[1], Zhihao Yu[1], Xiaoli Zhang[1], Shougang Fan[1], Huiyu Gao[1], Caini Liu[1], Qixing Zhou[1], Huaiqi Shao[2], Lan Wang[1]，Xiaoyan Guo[1]**

[1] Ministry of Education Key Laboratory of Pollution Processes and Environmental Criteria, Tianjin Key Laboratory of Environmental Technology for Complex Trans-Media Pollution, Tianjin Key Laboratory of Environmental Remediation and Pollution Control, College of Environmental Science and Engineering, Nankai University, No. 38 Tongyan Road, Haihe Education Park, Jinnan District, Tianjin 300350, China;

[2] College of Material Science and Chemical Engineering, Tianjin University of Science & Technology, No.29 Thirteenth Street, TEDA, Tianjin 300457, China

Corresponding Author and Address:

Xiaoyan Guo

E-mail: guoxyan@nankai.edu.cn

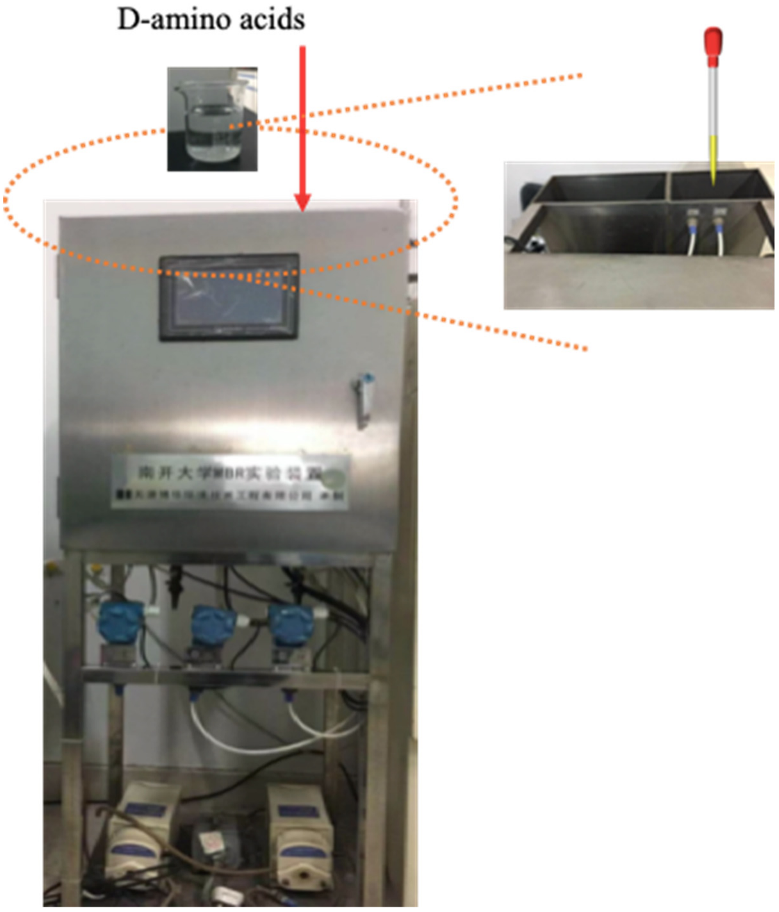

Figure S1 Real photo of MBR

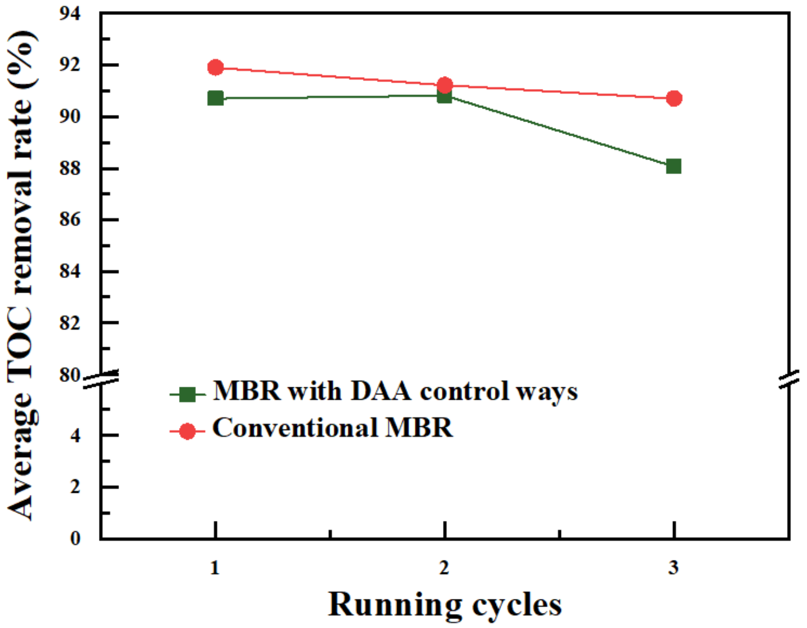

Figure S2 Average TOC removal rate between conventional MBR and MBR with DAA control ways

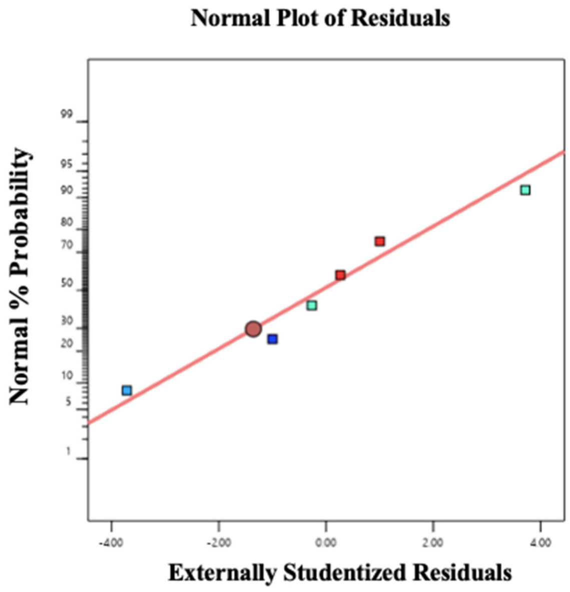

Figure S3 The relationship between internally studentized residuals and normal % probability.

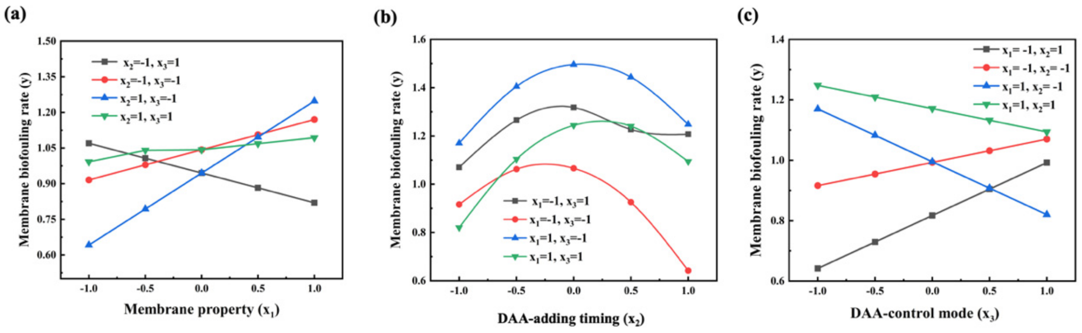

Figure S4 The prediction of membrane biofouling rate (y) varied with (a) membrane property (x_1), (b) DAA-adding timing (x_2) and (c) DAA-control mode(x_3) under different combinations of DAA control ways, respectively. In which, x_1 = -1 for hydrophilic membrane; x_1 = 1 for hydrophobic membrane; x_2 = -1 for adding DAA before running; x_2 = 1 for adding DAA after running; x_3 = -1 for adding DAA as cleaning agent; x_3 = 1 for adding DAA as active agent.

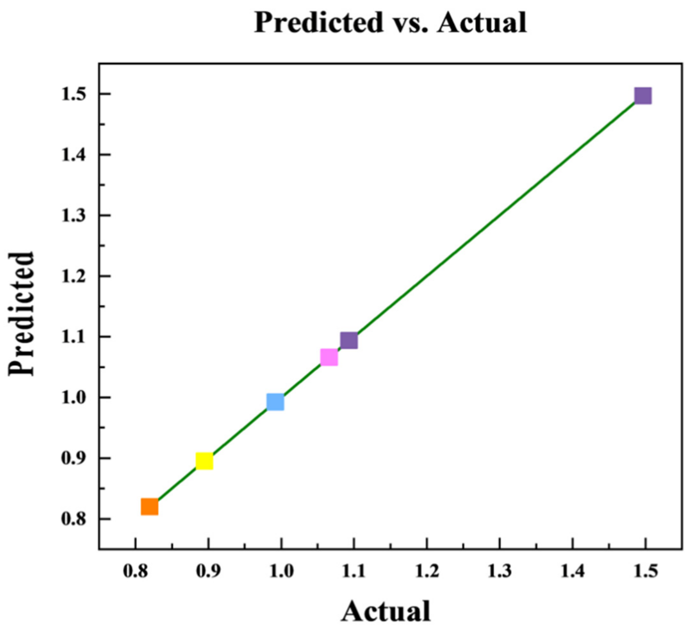

Figure S5 Fitting of simulated value and measured value

Table S1 Composition of synthetic municipal wastewater.

Components	Content
Sodium acetate	431.25mg/L
Glucose	195.63mg/L
NH_4Cl	125mg/L
K_2HPO_4	37.5mg/L
$CaCl_2$•$2H_2O$	9.375mg/L
$MgSO_4$•$7H_2O$	7.8125mg/L
$FeSO_4$•$7H_2O$	12.5mg/L

Table S2 Operation parameters for the MBR.

Operational Parameters	Values
Working Volume (L)	43
Temperature (°C)	25 ± 2
pH	7.0-7.5
Hydraulic Retention Time (HRT) (h)	10
Permeate Flux ($L \cdot m^{-2} \cdot h^{-1}$)	1.2
Aeration Rate ($L \cdot min^{-1}$)	40
MLSS ($mg \cdot L^{-1}$)	4900-5100
Suction Pump Speed (rmp)	10
Pumping Time (min)	8
Stopping Time (min)	2
D-tyrosine Concentration ($mg \cdot L^{-1}$)	6

Table S3 The control protocols of DAA in MBR.

Experiment batches	Operational parameters		
	Membrane property	DAA-adding timing	DAA-control mode
1	**Hydrophilic PAN membrane**	Before running	Active agent
2	**Hydrophobic PVDF membrane**	Before running	Active agent
3	Hydrophilic PAN membrane	**Before running**	Active agent
4	Hydrophilic PAN membrane	**After running**	Active agent
5	Hydrophilic PAN membrane	After running	**Active agent**
6	Hydrophilic PAN membrane	After running	**Cleaning agent**

Table S4 Membrane biofouling rate of MBR with DAA control way and conventional MBR during three cycles.

Device	Biofouling-physical cleaning cycle			Average membrane biofouling rate
	one	two	three	
MBR with DAA control way	0.6650	1.0981	1.4348	1.0660
Conventional MBR	1.0621	1.4301	1.6236	1.3719

Table S5 Independent variable regression coefficients (t-test) and analyses of variance (ANOVA).

Model	R^2	Unstandardized Coefficients		Standardized Coefficients	Sig.	Collinearity statistics	
		B	Std. Error	Beta		Tolerance	VIF
(constant)	1.000	1.069	0.000		0.000		
x_1		0.089	0.000	0.411	0.000	0.667	1.500
x_3		- 0.075	0.000	- 0.328	0.000	0.375	2.667
x_1x_2		0.088	0.000	- 0.584	0.000	0.667	1.500
x_1x_3		- 0.126	0.000	0.407	0.000	0.167	6.000
x_2x_3		0.049	0.000	0.212	0.000	0.130	2.667

Table S6 Membrane biofouling rate r_p varied with different DAA control ways in every biofouling-physical cleaning cycle.

Experiment batches	Operational parameters			Membrane biofouling rate r_p
	Membrane property (-1 for hydrophilicity;1 for hydrophobicity)	DAA-adding timing (-1 for before `running;1 for after running)	DAA-control mode (-1 for cleaning agent;1 for active agent)	
1	-1	1	-1	1.0660
2	1	1	-1	1.4969
3	-1	-1	1	0.8948
4	1	-1	1	0.8198
5	-1	1	1	0.9921
6	1	1	1	1.0933

Table S7 Pearson correlation coefficients between membrane biofouling rate (r_p) and DAA control ways (x_1, x_2, x_3).

r_p	x_1	x_2	x_3	x_1x_2	x_1x_3	x_2x_3	x_1x_1	x_2x_2	x_3x_3
Pearson Correlation	0.352	0.663	-0.721	0.730	-0.311	-0.663			
Sig.	0.025	0.005	0.008	0.050	0.027	0.008	0.000	0.000	0.000
Number of samples	6	6	6	6	6	6	6	6	6

Table S8 DAA control ways optimization combination solution sorted for r_p.

Number	Membrane property (-1 for hydrophilicity;1 for hydrophobicity)	DAA-adding timing (-1 for before running;1 for after running)	DAA-control mode (-1 for cleaning agent;1 for active agent)	r_p	Desirability
1	1	-1	1	0.820	1.000 (selected)
2	-1	-1	1	0.895	1.000 (selected)
3	0.972	-1	1	0.821	0.998
4	1	-0.986	1	0.822	0.997
5	1	-0.977	1	0.823	0.995
6	1	-0.969	0.998	0.825	0.993
7	1	-0.956	1	0.826	0.991
8	1	-1	0.974	0.827	0.989
9	1	-0.937	1	0.828	0.987
10	0.692	-1	1	0.831	0.983

***The first 10 solutions exhibited in Table S8.**

Table S9 Detailed simulation values (random pick) of continuous membrane biofouling under different DAA control ways.

Number	Membrane property (-1 for hydrophilicity;1 for hydrophobicity)	DAA-adding timing (-1 for after running;1 for before running)	DAA-control mode (-1 for cleaning agent;1 for active agent)	Membrane biofouling rate (r_p)
1	1	1	-1	0.8198
2	-0.6	0.4	0.2	1.0212
3	-1	1	1	0.9921
4	0.06	-0.16	0.56	1.1850
5	-1	1	-1	0.8948
6	0.38	-0.76	0.14	1.3293
7	-1	-1	-1	1.0174
8	-0.9	0.72	0.84	1.0088
9	1	-1	1	1.6337
10	0.5	-0.08	0.64	1.2621
11	-1	-1	1	1.1147
12	0.88	0.76	1	1.1491
13	1	-1	-1	1.3602
14	1	1	1	1.0933
